# African fermented foods: overview, emerging benefits, and novel approaches to microbiome profiling

**DOI:** 10.1038/s41538-022-00130-w

**Published:** 2022-02-18

**Authors:** Yemisi D. Obafemi, Solomon U. Oranusi, Kolawole O. Ajanaku, Paul A. Akinduti, John Leech, Paul D. Cotter

**Affiliations:** 1 Department of Biological Sciences, Covenant University, Ota, Ogun State Nigeria; 2 Department of Food Biosciences, Teagasc Food Research Centre, Moorepark, Fermoy, Ireland; 3 Department of Chemistry, Covenant University, Ota, Ogun State Nigeria; 4 APC Microbiome Ireland, Cork, Ireland; 5 VistaMilk Research Centre, Cork, Ireland

**Keywords:** Applied microbiology, Microbiome, Metagenomics

## Abstract

Traditional fermented foods are of major importance with respect to the socio-economic growth, food security, nutrition, and health of African consumers. In several African countries, traditional fermentation processes provide a means of food preservation, improving the shelf life and adding to the nutrients in the food products. As with any fermented foods, the associated food microbiota is of great importance and interest. Recent studies on the microbiome of African fermented foods using high-throughput DNA sequencing techniques have revealed the presence of diverse microbial populations of fundamental, technological, and commercial interest that could be harnessed to further improve health, food safety, and quality. This review provides an overview of African fermented foods, their microbiota, and the health-promoting potential of these foods and microbes.

## Introduction

Fermented foods play an essential role in tackling issues of poverty, malnutrition, and hunger among African consumers^1^. These foods also enhance food security, sustainable development, and economic growth in Africa such as through the provision of employment opportunities, contribution to empowerment initiatives for unemployed women, opportunities for scaling up of traditional food processing techniques, and distribution of resultant products^2^. Fermented foods can be a rich source of nutrients, with fermentation bringing about a “pre-digestion” of food substrates to make the associated nutrients more bioavailable and, in some instances, removing allergens (including 2S albumin proteins, profilins, cupin, prolamine), antinutritional compounds (such as phytate, tannins, lectins, protease inhibitors, saponins, alkaloids, and oxalate) and toxins (such as cyanogenic glycosides, bacterial toxins, mycotoxins, biogenic amines)^3^. The associated microbes can have health**-**promoting properties either directly, with some probiotic strains from most commonly consumed and extensively investigated fermented foods in Africa or indirectly, through the production of health-promoting metabolites. Several fermented foods also contain prebiotic components^4^. Indigenous fermentation processes represent traditional, reliable, and affordable methods of preserving nutritional and sensory qualities of associated substrates while extending shelf life and enhancing safety. Fermentation of these foods in several African countries usually involves spontaneous activities of natural microbes found in the food or from a previous fermentation, such as through “back-slopping” to bring about the desirable changes^5^. Fermentation of certain foods facilitates wet milling, the aforementioned removal of toxic or anti-nutritive compounds, and improvement of organoleptic properties and extension of the shelf life of the food products. The preservative effects of fermented foods are particularly important in Africa considering the tropical climatic conditions across large regions of the continents. The climate makes the preservation of fresh farm products such as milk, fruits, and vegetables very challenging, resulting in reports of over 70% loss of some products yearly^6,7^. Post-harvest spoilage of fresh foods is usually enhanced by environmental pollution and contamination which results in physical rotting, esthetic spoiling of the environment, and bad odor, ultimately making the food unsuitable for consumption. Although the preferred approach for the preservation of fresh foods is refrigeration, inadequate power supplies and low living standards in certain regions of Africa limit this option^8^. Thus, in some instances, food fermentation is the only option for preserving foods, resulting in products with enhanced acceptability, digestibility, functionality, and nutritional quality.

Such enhancements are more controllable where there is a corresponding understanding of the microbiota of the fermented food. Thus, while, to date, there is a corresponding understanding of many of the functional pathways and metabolic potential of several fermenting genera including *Leuconostoc*, *Lactobacillus*, *Pediococcus*, *Debaryomyces*, *Kluyveromyces*, *Saccharomyces*, etc^9–14^, this is increasingly complemented by advances in shotgun metagenomic sequencing that, in addition to identifying the microorganisms present (including novel species and strains), can provide an insight into potential metabolic and functional pathways encoded^15–18^.

### Commonly consumed fermented food products in Africa

**Fig. 1:**
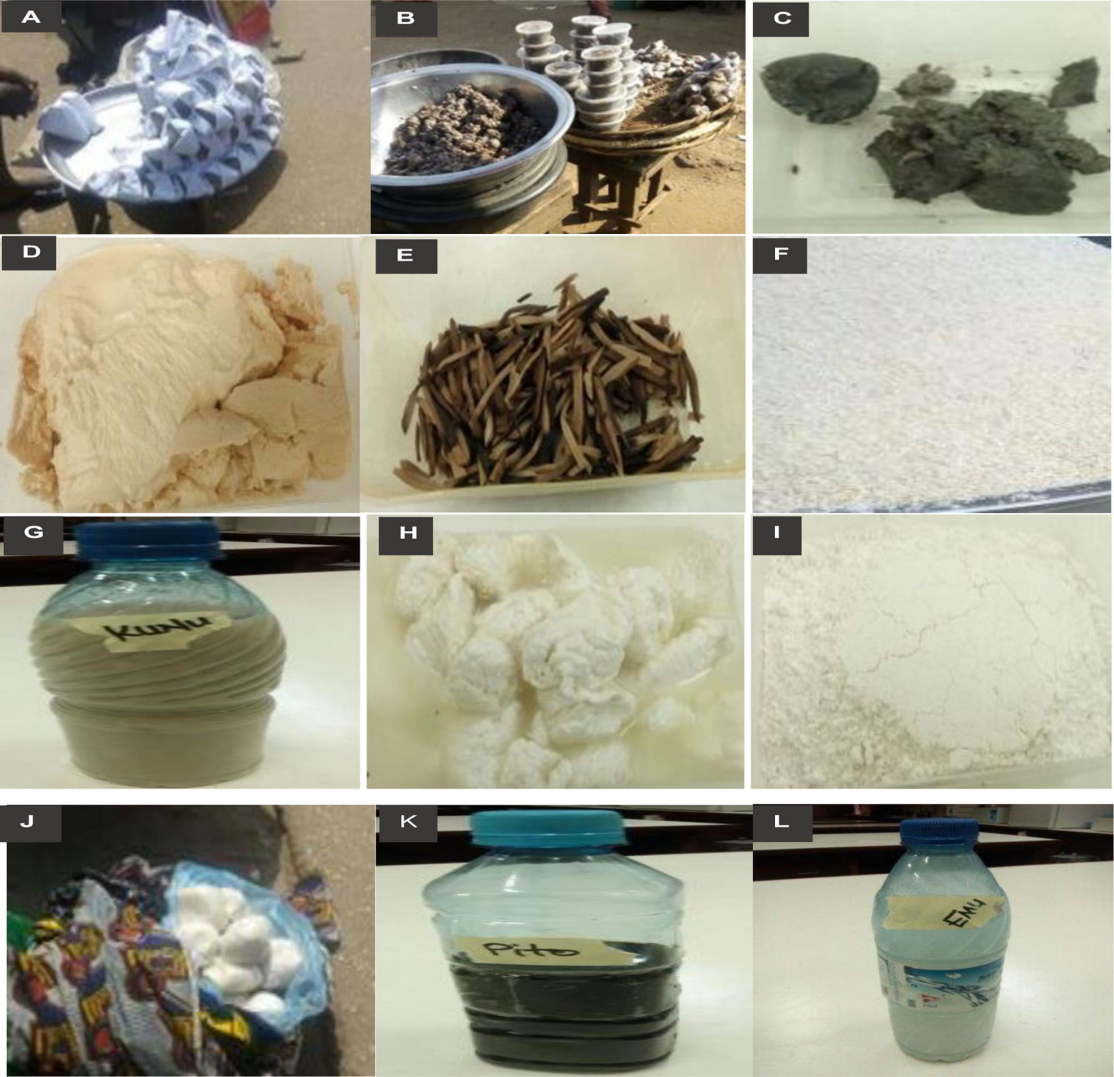
Common fermented foods in Africa (**A** = Eko, **B** = Iru, **C** = Ogiri, **D** = Ogi, **E** = Ugba, **F** = Gari, **G** = Kunu, **H** = Wara, **I** = Lafun, **J** = Fufu, **K** = Pito, **L** = Emu).

Fermented foods represent a significant component of the diverse range of African diets, with individual foods being assigned, for example, as staple foods, condiments, or beverages. The foods can also be classified according to the raw materials used for their production. These include tuber-based products (e.g., “Fufu,” “Lafun,” and “Gari”); cereal-based products (e.g., “Ogi,” “Burukutu,” “Kunnu-zaki,” and “Pito”); legume-based products (e.g., “Iru” or “Dawadawa,” “Ogiri,” and “Ugba”); beverage-based products (e.g., “Emu,” “Oguro”), and dairy-based products (e.g., “Fura,” “Nunu,” “Wara-Kishi”) (Fig. 1). These respective categories are described in greater depth below.

### Fermented tuber products

Tubers such as cassava (*Manihot esculenta*), yam (*Dioscorea alata*), and sweet potato (*Ipomoea batatas*) are common starchy foods. They are rich in carbohydrates and are generally cultivated for consumption in individual African households. Government policies in many African countries also support the production of cassava and fermented cassava products as an alternative to imported wheat and rice products^19^. Fermented cassava products commonly consumed across African countries include those produced from the tubers (fresh or dried), cassava leaves, milled paste or granulated fried products^20^. Tubers are usually peeled, washed, milled, and fermented for about 3–5 days into sour granulated flakes called “Gari” (Nigeria), steamed granulated flakes “Attieke” (Côte d’Ivoire) or milled into flour called “Lafun” or “Kokonte” (Nigeria and Ghana) to prepare meals such as “Amala,” “Eba,” and “Fufu” (Nigeria). Other fermented tuber products include “Agbelima” (Ghana), “Atangana,” “Kumkum,” “Myiodo” (Cameroon), “Maphumu,” and “Makaka” (Malawi)^21–23^. The hydrogen cyanide (HCN), linamarin, loutaustralin, and other undesirable compounds in cassava are eliminated during processing which includes peeling, grating, soaking, pressing, and frying/cooking^24^. The cyanogenic glycosides are hydrolyzed by beta-glucosidases and HCN evaporates prior to consumption of the products^25^. Fermentation further eliminates the cryogenic glycosides through microbial activities (such as that by lactic acid bacteria (LAB) and yeasts) generating metabolites, including organic acids and volatile compounds, which contribute to the distinct organoleptic properties of the fermented products^26–29^. Several LABs have been isolated from African fermented tuber food products and found to produce antimicrobial agents, vitamins, and other compounds of interest. Microorganisms isolated from fermented tuber products include representatives of the species: *Lactobacillus acidophilus*, *Levilactobacillus brevis*, *Limosilactobacillus fermentum*, *Ligilactobacillus salivarius*, *Limosilactobacillus reuteri*, *Lacticaseibacillus casei*, *Enterococcus faecium*, *Bacillus subtilis*, *Geotrichum candidum*, *Mucor racemosus*, *Saccharomyces cerevisae*, *Neurospora sitophila*, and *Rhizopus oryzae*^30–32^.

### Fermented cereal products

Cereal fermented porridges or beverages with live and active microorganisms are much more common in Sub-Sahara Africa than in other places of the world. The food products form major components of staple foods, used as a part of weaning diets for infants and essential functional foods for children. The foods are produced from millet (*Panicum miliaceum*), wheat (*Triticum aestivum*), maize (*Zea mays*), rice (*Oryza sativa*), and sorghum (*Sorghum bicolor*) and, as with other fermented foods, they undergo biochemical, and nutritional changes during the fermentation process^33,34^. The fermented cereal products can be in the form of a paste or slurry as in the case for “Ogi” (Nigeria), “Akasa” (Ghana), “Uji” (Kenya), “Abreh” (Sudan), “Mahewu” (Zimbabwe and South Africa) including fermented drinks like “Burukutu” (Nigeria) and “Kunnu” (Nigeria)^35,36^. The cereal-based fermented foods are reported as good sources of beneficial microorganisms, micronutrients and are regarded as health-promoting foods^37–39^. Fermented cereal products also contain LAB that contributes extensively to nutritional properties as well as to food safety, shelf-life quality, and organoleptic properties through the production of organic acids, bacteriocins, and volatile compounds. These LABs include *Lactiplantibacillus plantarum*, *Limosilactobacillus fermentum*, *Levilactobacillus brevis*, *Lactococcus lactis*, *Leuconostoc* species, and *Pedicoccus* species, while the other bacteria represented include *Streptococcus* and *Corynebacterium* species. Yeasts such as *Saccharomyces cerevisiae*, *Geotrichum fermentum*, *Candida tropicalis*, *Rhodotorula graminis*, and molds such as *Aspergillus species*, *Fusarium subglutinans*, *Rhizopus nigricans*, and *Penicillum citrinum* have been reported to be present at the early stage of the fermentation of these products (Table 1)^36,37,40^.

**Table 1:** Occurrence of various fermenting microorganisms among commonly consumed selected African fermented foods.

Substrate	Fermented food	Microorganisms	Origin	Ref
Maize, sorghum millet	Mawè	*L. fermentum*, *L. brevis*, *L. curvatus*, *L. buchneri*, *Weissella confusa*, *Pedicoccus pentosaceus*, *Candida krusei*, *Candida kefyr*, *Candida glabrata*, *Saccharomyces cerevisiae*	Benin	^65^
Maize, sorghum millet	Uji, Ogi	*L. plantarum*, *L. fermentum*, *L. cellobiosus*, *L. buchneri*, *Ped. acidilactici*, *Ped. pentosaceus, S. cerevisiae, Rhodotorula graminis*, *Candida krusei*, *Candida tropicalis*, *Geotrichum candidum*, *Geotrichum fermentum*	Benin, Nigeria	^39,65^
Millet	*Ben-saalga*	*L. fermentum*, *L. plantarum*, *Ped. pentosaceus*	Burkina Faso	^65^
Cassava	Gari	*L. fermentum*, *L. plantarum*, *W. confusa*, *S. cerevisiae*, *Pichia scutulata*, *Kluyveromyces marxianus*, *Hansenia guilliermondii*, *Leuconostoc fallax*	Nigeria	^65^
Milk	*kule naoto* *Amasi* *Ergo*	*L. fermentum*, *L. paracasei*, *L. acidophilus*	Kenya South Africa Zimbabwe	^49,51,52,77^
Maize, sorghum millet	Mahewu (magou)	*L. delbrueckii* subsp. *bulgaricus*; *L*. *delbrueckii* subsp. *delbrueckii*; *Leuconostoc* spp.; heterofermentative lactobacilli	South Africa	^65^
Millet, sorghum	Kunu-zaki	*L. fermentum*, *Ped. pentosaceus*, *W. confusa*, *Ent. faecalis*	Nigeria	^36^
Cassava	Fufu	*Ped. pentosaceus*, *L. fermentum, L. plantarum*	Nigeria	^65^
Cassava	Agbelima	*L. plantarum*, *L. brevis, L. fermentum*, *Leuc. mesenteroides*, *Bacillus* spp., *C. tropicalis*, *G. candidum*, *Penicillium* spp.	Ghana	^65^
Cow milk	Mafi	*Lc. lactis subsp. lactis*; *L. delbrueckii* subsp. *lactis*; *L. plantarum*, *Leuc. mesenteroides* subsp*. dextranicum*	Namibia	^52^
Cow milk	Leben	*Lc lactis* subsp*. lactis*; *Lc. lactis* subsp*. Lactis biovardiacetylactis*; *Kluyveromyces lactis*, *S. cerevisiae*, *E. faecalis*, *L. brevis*, *Lc. lactis* subsp*. cremoris*; *Leuc. lactis*, *Leuc. mesenteroides* subsp*. dextranicum*	Morocco	^52^
Cow milk	Amabere amaruranu	*L. fermentum*, *L. paracasei*, *L. acidophilus*, *Lc. lactis* subsp*. lactis*; *S. cerevisiae*, *E. faecalis*, *Lb. brevis*, *Lc. lactis* subsp*. cremoris*	Kenya	^54^

### Fermented legume products

African fermented legume products are important sources of proteins, which are made more bioavailable following microbial digestion and detoxification, thereby contributing to countering malnutrition. These foods are often produced as condiments but have not been successfully commercialized on a large scale due to their short shelf-life and challenges with achieving packaging standards for the finished products and, so, continue to be produced using traditional methods^41,42^. The fermented condiments of plant-based proteinaceous foods include; “Iru” or “Dawadawa,” “Tempeh,” “Soumbala,” and “Netetu” from African locust beans, (*Parkia biglobosa*) (Nigeria, Ghana, Burkina Faso, Senegal), “Ugba” from African oil beans (*Pentaclethra macrophylla*) (Nigeria, Benin), “Ogiri” from either of castor oil beans (*Ricinus Communis*), Melon seeds (*Citrullus vulgaris*) (Nigeria), Fluted pumpkin seeds (*Telfairia occidentalis*) (Nigeria), or Bambara groundnut seeds (*Vigna subterranean*) (Nigeria), “Okpiye” from Prosopis Africana seeds (*Prosopis africana*), “Owoh” from African yam beans (*Sphenostylis stenocarpa*) (Nigeria) and “Siljo” from Faba beans (*Vicia faba*) flour mixed with safflower (*Carthamus tinctorius*) (Ethiopia). Fermented condiments are produced by the alkaline fermentation of legume seeds and are associated with abundance of *Bacillus subtilis*, *Bacillus megaterium*, *Bacillus circulans*, *Bacillus licheniformis*, and *Bacillus coagulans*^43–45^. Other bacteria identified previously included strains of *Lactiplantibacillus plantarum*, *Lb. acidophilus*, *Lb. delbrueckii* subsp*. bulgaricus*, *Yarrowia lipolytica*, *Saccharomyces cerevisiae*, *Saccharomyces rouxii*, *Rhodotorula glutinis*, *Staphylococcus aureus*, *Escherichia coli*, *Streptococcus natalensis*, *Proteus* species, *Alcaligenes* species, *Micrococcus*, *Corynebacterium*, *Enterococcus faecium*, and *Pseudomonas* species^46,47^.

### Fermented dairy products

Fermented dairy products are often produced through the fermentation of milk. Dairy products such as “Nunu” (Nigeria, Ghana), “Wara” (Nigeria, Togo), “Fene” (Côte d’Ivoire, Mali), “Suusac” (Kenya, Somalia), “Pendidam” (Cameroon), “Gariss” (Sudan), “Nyamie” (Ghana), “Leben/lben” (Tunisia) and “Kulenaoto” (Kenya) are important fermented dairy products of considerable nutritional importance providing macronutrients, micronutrients, and health-promoting microorganisms. Such dairy fermented foods are normally introduced by backslopping of previously fermented dairy products to serve as a starter culture for a new fermentation. Fermented dairy products are widely consumed by Africans due to the health-promoting benefits linked to the microbial contents of the foods and the presence of specific micronutrients^48^. Various researchers have documented the dominance of LABs in African fermented milk, including strains of *Lb. delbrueckii* subsp. *bulgaricus*, *Lb. acidophilus*, *Lactococcus lactis* subsp. *lactis*, *Lactococcus lactis* subsp*. cremoris*, *Limosilactobacillus fermentum*, *Ligilactobacillus salivarius*, *Lactiplantibacillus plantarum*, *Enterococcus*, and *Streptococcus* species (Table 1)^48–51^. Other microorganisms including *Staphylococcus* species, *Streptococcus infantarius* subsp. *infantarius*, *Trichosporon* species, *Saccharomyces cerevisae*, *Rhodotorula mucilaginosa*, and *Geotrichum penicillatum* have also been identified. Notably, several researchers have reported that the presence in dairy products of strains corresponding to many of the taxa listed above, introduced as starter cultures or as part of the resident microbiota, is associated with improved organoleptic, nutritional safe, and shelf-life qualities^48,52^.

### African fermented foods as a “storehouse” for potential health-promoting microorganisms

As noted above, there is a wide range of locally produced fermented foods and beverages across Africa. This results from a wide variety of fermentation types (lactic acid, alcohol, alkaline fermentation), substrates, microorganisms, and methods. Although food fermentation in Africa mostly occurs through spontaneous fermentation, there are also instances where the microorganisms were introduced by backslopping from previously fermented foods into new fermentations^53^. African fermented foods have been reported to be sources of many potentially nutritionally beneficial and/or health-promoting components, including microorganisms. Unsurprisingly, from a microbial perspective, there has been a particular focus on members of the lactic acid bacteria such as members of the former genus *Lactobacillus*^54^ (e.g., from fermented condiments “Iru,” “Ugba,” and “Ogiri” and fermented cereal-based foods “Ogi”), *Lactococcus* species (e.g., from fermented milk “Nunu” and yoghurt “Wara”) *Pediococcus* species and *Weissella* species (e.g., from fermented cereal-based foods “Ogi” and “Kunun”)^55,56^. We use the word “potentially” with care, as many indications of health benefits do not match the criteria required to use the term probiotic^57^. Further investigations are needed (including clinical trials) before the term probiotic can be used in the context of these microorganisms.

Despite this, the data that is available in relation to the health-promoting properties of specific African fermented foods and their associated microorganisms provides cause for optimism, with potential future applications including preventing the colonization of pathogens, treatment of infectious diseases, improved lactose tolerance, immune system modulations, improved barrier function, interference with quorum sensing signaling and the production of antimicrobial substances^58^. There are, however, limited reports on clinical randomized trials on the application of African fermented foods to improve general health with positive outcomes on conditions such as acute diarrhea in infants^59–61^. This could be associated with the microbiota in controlling enteric pathogens by inhibiting attachment of the pathogens’ pili to gut mucosa, through the release of organic acids and antimicrobial peptides, by competing for limited nutrients as well as the neutralization of exotoxins released by pathogens^62,63^.

The gut microbiome is now increasingly becoming a target with a view to improving human health including, in its most extreme form, the use of fecal microbiome transplants for the treatment of *Clostridioides difficile* infection^64^. However, from a practical “every day,” perspective, dietary modulation of the human microbiome is a more appealing alternative. Fermented foods were a particularly popular option in this regard, with the potential for modulation of the host microbiome (or host cell activity) by components of the foods (i.e., microbes, metabolites, digested components of the original substrate). Studies showing consumption of fermented foods is both correlated with, and responsible for, improved health outcomes^65^. Despite this, fermented foods and associated microbes remain surprisingly relatively underexplored, particularly in respect to potential health benefits and modulation of the human microbiome^66^. African fermented foods are especially underexplored in this respect^67^. Fundamentally, African fermented food research provides researchers with novel insights into underexplored foods (and associated microbiota), but applying the research can also be beneficial for empowering local non-scientific producers of the food with prospects for commercialization and health-promoting benefits^68–70^.

### Health-promoting potentials of bioactive components in African fermented foods

The cereal-based African fermented foods (rice, millet, maize, or sorghum) are rich in bioactive substances released during fermentation but also contain anti-nutritional factors including polyphenols, tannins, and phytic acid, which reduce the digestibility and bioavailability of carbohydrates and proteins. These foods can also serve as a source of prebiotics, which are “substrates that are selectively utilized by host microorganisms conferring health benefits”, which include non-digestible dietary fractions, such as oligosaccharides, polysaccharides, resistant starches, polyphenols, and resistant proteins^71^. Microbial hydrolysis of certain prebiotics has also been reported to reduce anti-nutritional factors and thus improve the nutritional profile of the food products. Fermented cereal foods (such as “Ogi,” “Mahewu,” “Incwancwa,” “Togwa,” “Borde,” or “shamita”) made from maize were previously reported to be a very rich source of vitamin B complex including folic acid, pyridoxine, pantothenic acid, thiamine, and riboflavin while fermented sorghum-based foods (such as “Munkoyo,” “Uji,” “Ogi,” “Kunu”) are rich in amino acids such as alanine, aspartic acid, glutamic acid, leucine, phenylalanine proline, and valine with low tryptophan and lysine. Some studies have also reported the presence of trace amounts of β-carotene^72,73^.

### Microbial profiling techniques and the microbiota of African fermented foods

In recent years, high-throughput sequencing (HTS) techniques have been used to an increasing extent to study the microbiome of fermented foods from across the globe, including gaining insights into microbial dynamics, food safety, and putative health benefits^74^. Despite the increasingly widespread use of these approaches, the study of fermented foods in Africa still relies heavily on the use of classical culture-based methods combined with some polymerase chain reaction (PCR)-based techniques. Other population-based analyses such as those involving DGGE (denaturing gradient gel electrophoresis) have also been used in the past^75^ but do not facilitate the easy identification of the taxa present. In addition, the use of DGGE provides a dependable and considerable profile of diverse bacteria communities in cereal-based fermented foods (Amasi) commonly consumed in south Africa^76^ and PCR-DGGE fingerprints of microbial successional changes during fermentation of cereal-legume weaning foods in Nigeria^77^. Currently, Rep-PCR fingerprinting, 16S and 23S rRNA gene sequencing techniques, shotgun metagenomics sequencing, and whole-genome sequencing are infrequently used to study the microbial ecology of African fermented foods, and the microbial species/strains that are present therein. This infrequent use reflects the lack of access to the appropriate infrastructure and the high cost of applying HTS techniques to study the microbiomes and genomic diversity of strains in Africa fermented foods^78,79^.

Frequently, the methods used to analyze fermented foods in Africa precluded the characterization of non-culturable, or difficult to culture microbes, and thus specific components of microbial populations in these food ecosystems may have been overlooked. Although some recent studies have tried to address this problem by developing modified DNA extraction protocols for subsequent genomic and metagenomic sequencing of selected African fermented foods such as “Dengue” (Burkina-Faso), “Lafun” (Nigeria), “Ogi” (Nigeria), “Masau” (Zimbabwe), “Obusera” (Uganda), “Fura” (Ghana), the full potential of HTS has yet to be widely applied to the study of African fermented food microbiomes^80–83^.

### Potential application of HTS techniques for commonly consumed African fermented foods

Advances in microbial analysis of the food ecosystem have involved the use of HTS techniques such as whole-genome sequencing, amplicon bases meta-taxonomic approaches (such as 16S rRNA sequencing), shotgun metagenomics, and (meta) transcriptomics, and rely on downstream bioinformatics analysis^84^. In Africa, whole-genome sequencing was initially used for the analysis and surveillance of foodborne pathogens. This process supplements other pre-existing methods such as culture-based isolation, bio-typing, serotyping, genotyping, as well as virulence and antimicrobial gene detection for food-associated pathogenic microorganisms^85,86^. As discussed, this technique could also be harnessed to assess food isolates from the perspective of their value as a starter or adjunct cultures by identifying genotypic traits underpinning features such as the production of bacteriocins or other antimicrobials of relevance from most consumed African fermented foods for improved food preservation and shelf-life quality as listed in Table 2. These tools could be applied to screen for pathways associated with improving the nutritional quality of the food and/or imparting other health-promoting characteristics.

**Table 2:** Previous studies on the microbial analysis of predominantly consumed African fermented foods evaluated with various sequencing platforms.

Fermented foods	Raw materials	Country of study	Year of study	Methodology used	References
Masau	Masau fruits	Zimbabwe	2017	16S HTS	^83^
Mursik	Milk	Kenya	2017	Sanger sequencing	^89^
Nunu	Milk	Ghana	2017	Shotgun sequencing	^17^
Fura	Millet	Nigeria	2019	16S HTS	^81^
Wara	Milk	Nigeria	2019	16S HTS	^73^
Kunun	Sorghum	Nigeria	2019	16S HTS	^36,85^
Kokonte	Cassava	Ghana	2019	16S HTS	^72^
Gari	Cassava	Ghana	2019	16S HTS	^72^
Mawe	Maize	Benin	2019	16S HTS	^72^
Sombana	Milk	Burkina Faso	2019	Sanger sequencing	^74^
Maari	Milk	Burkina Faso	2019	Sanger sequencing	^74^
Mahoto	Sorghum	South Africa	2019	16S HTS	^72^
Teff dough	Teff	Ethiopia	2019	16S HTS	^47^
Tej	Honey	Ethiopia	2019	16S HTS	^69^
Tonton	Banana	Uganda	2019	16S HTS	^69^
Wagashi	Milk	Benin	2020	Shotgun sequencing	^18^
Kisra	Sorghum	Sudan	2020	Sanger sequencing	^87^
Hulumur	Sorghum	Sudan	2020	Sanger sequencing	^87^

The 16S rRNA gene amplification combined HTS allows for genus level assessment of the microbiota and technological improvement is constantly reducing sequencing costs, resulting in affordability of HTS in diverse research applications. Shotgun metagenomic sequencing facilitates deeper insights into the microbiome, allowing strain-level identification, functional annotation including carbohydrate pathways and bioactive molecule production (such as bacteriocins), and the assembly of high-quality genomes in the form of metagenome-assembled genomes (MAGs)^18,87^. In the latter case, the calculation of Average Nucleotide Identity scores of newly assembled MAGs with existing genome databases facilitate taxonomic assignment at species and even at strain level^88^.

**Fig. 2:**
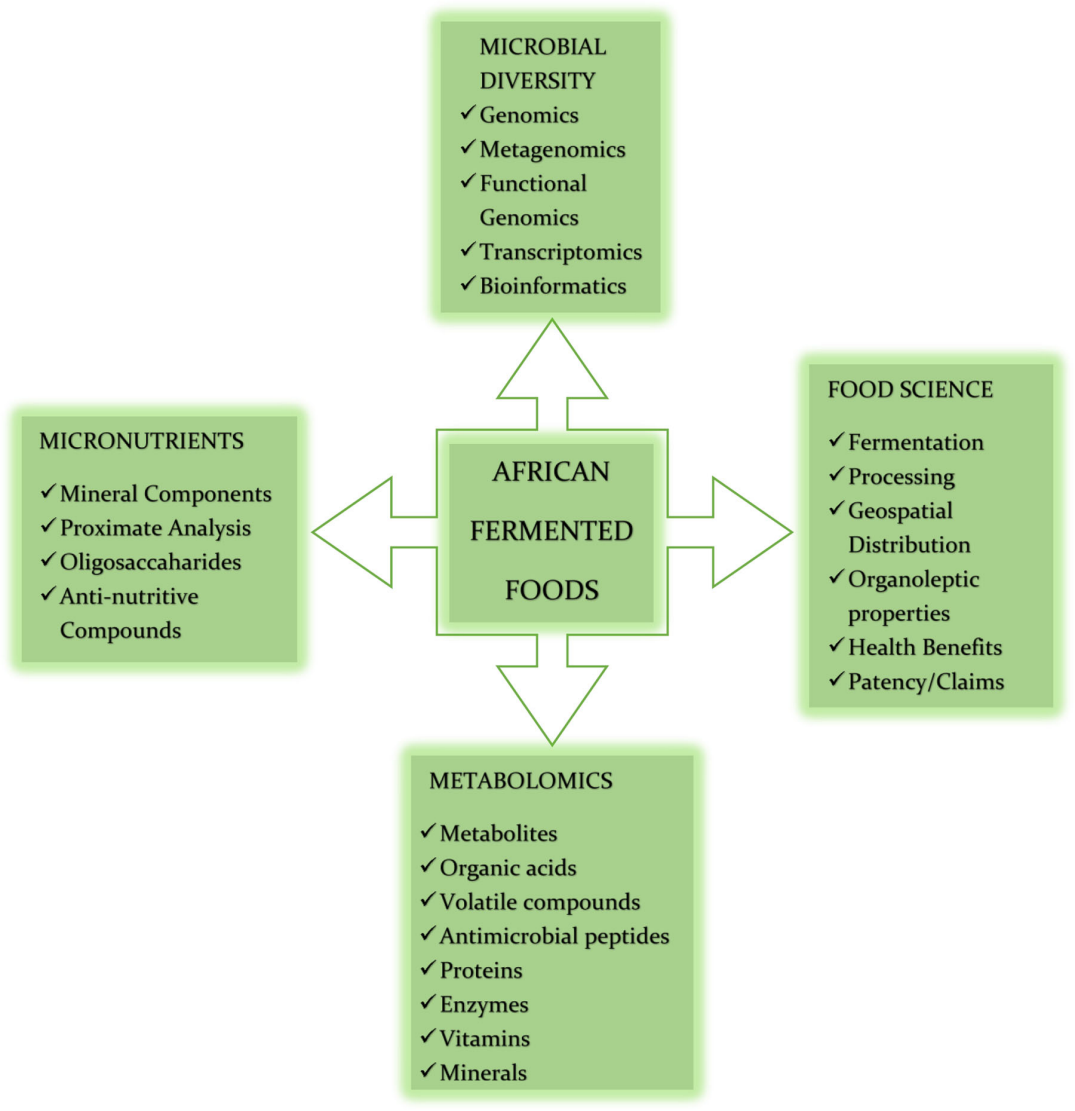
Overview of potential study areas in African fermented foods.

Several HTS sequencing platforms, such as those developed by Illumina, Ion Torrent, PacBio, BGI, and Oxford Nanopore technologies and protocols could reveal microbial taxonomy, diversity, functional potential, and/or gene expression patterns with different results. The Illumina sequencing platforms have been widely employed for the generation of extremely high quality, short reads of up to 2×300 bp. Conversely, MinION platform based on single-molecule sequencing technology generates considerably longer read lengths but with lower accuracy and read numbers than short-read HTS platforms. Short read sequencing is not capable of completely closing bacterial genomes from mixed communities while using long-read data with a high error rate has other associated drawbacks, such as, for example, an inability to reliably identify single-nucleotide polymorphisms. However, by combining both approaches for whole-genome sequencing, fully assembled chromosomes and plasmids can be recovered with low error rates^89^. As DNA sequencing technologies continue to improve, the use of existing techniques inevitably becomes more cost-effective, facilitating the more widespread use of these techniques globally. Indeed, access to HTS-based analyses is slowly becoming more accessible to African researchers interested in fermented foods and, as noted, it is hoped that this can contribute greatly towards the development of nutrient-based and fortified African fermented food^90^. As previously noted, the emergences of these innovations provide many possibilities for future research relating to commonly consumed African fermented foods shown in Fig. 2, and in particular, the development of microbial cultures for optimized food fermentations. Limitations of sequencing-based analyses still remain, such as live and dead cell differentiation (particularly important for spontaneously fermented foods), absolute count of microbes present (as opposed to relative abundance), and with some methods, limited taxonomic information and ambiguous assignment. The propidium monoazide-based sequencing methods are getting better at resolving live/dead cell differentiation, while metatranscriptomics can add a further layer of information in terms of which taxa are metabolically active. Flow cytometry and quantitative PCR can also be employed to facilitate insights into counts^91–98^. As noted above, combining long and short read data can facilitate the complete assembly of genomes including plasmids, mitigating some of the issues related to ambiguous assignment and false positives. However, sequencing-based analyses (particularly functional attributes) need validation by culture-based experiments.

### Microbial diversity profiling of African fermented foods ecosystem

For community-based investigations, amplicon sequencing of the hypervariable (V1–V4 or V4–V5) regions of the 16S rRNA amplicon gene and shotgun metagenomics, sequencing have improved insights into African fermented food products^17^. While fermented food products around the world are increasingly being studied using shotgun metagenomic techniques, very few studies have explored the use of shotgun sequencing in African fermented foods, with some exceptions being “Nunu” (Ghana), “Kokonte” (Ghana/Togo) and “Wagashi” (Benin)^17^. Leech et al. analyzed 58 fermented foods including “Nunu” (Ghana) and “Wagashi” (Benin) through shotgun metagenomic sequencing, which provided key insights into their microbial ecology and functional capabilities for health-promoting attributes^18^. Diaz et al. also investigated 40 African fermented foods using 16S rRNA metagenomic sequencing and found members of the genera *Lactobacillus*, *Leuconostoc*, *Weissella*, *Bacillus*, *Zygomonas*, *Streptococcus*, *Zymomonas*, *Gemmella*, *Brevibacillus*, *Jeotgalicoccus*, *Vagococcus*, *Atopstipes*, and others belonging to the family Carnobacteriaceae^79^. While these studies have highlighted the merits of applying HTS technologies to the study of African fermented food communities, there is a need for a broader-scale application.

## Conclusion

Fermented foods are a key component of the African diet and the associated microbes and microbial communities are being studied in ever-greater depth. The further harnessing of traditional culture-based and HTS techniques has the potential to characterize and optimize the microbiome of African fermented foods, from commercial to nutritional and health-promoting aspects. HTS can also be used in isolation, or in combination with cultivation, to identify virulence, antimicrobial resistance determinants, and provide genotypic characterization. Further access to HTS platforms and the democratization of sequencing have the potential to be of considerable value with respect to the further development of these foods.

## Data Availability

The authors confirm that all data are available within the article.
